# Lifestyle patterns in the Iranian population: Self- organizing map application

**DOI:** 10.22088/cjim.9.3.268

**Published:** 2018

**Authors:** Samaneh Akbarpour, Davood Khalili, Hojjat Zeraati, Mohammad Ali Mansournia, Azra Ramezankhanim, Akbar Fotouhi

**Affiliations:** 1 Department of Epidemiology and Biostatistics, School of Public Health, Tehran University of Medical Sciences, Tehran, Iran; 2 Occupational Sleep Research Center (OSRC), Baharloo Hospital, Tehran University of Medical Sciences, Tehran, Islamic Republic of Iran; 3 Prevention of Metabolic Disorders Research Center, Research Institute for Endocrine Sciences, Shahid Beheshti University of Medical Sciences, Tehran, Iran

**Keywords:** Lifestyle pattern, Self-organizing map, Cluster analysis, Lifestyle, Iran

## Abstract

**Background:**

The present study evaluated the lifestyle behavior patterns and its associations with demographic factors in the Iranian population.

**Methods:**

A total of 8244 people aged 25-70 years who participated in a national survey in 2011 were included in the study. Factors related to lifestyle (such as diet, physical activity, and tobacco use) have been collected using a questionnaire. A self-organizing map was used for cluster analysis and a multinomial logistic model was used for assessment of associations.

**Results:**

Seven clusters were identified as the following: cluster 1 (15.84%): healthiest lifestyle; cluster 2 (12.45%): excessive consumption of sweet tasting soft drinks, salt, and fast food; cluster 3 (33.73%): no recreational physical activity; cluster 4 (6.86%) alcohol consumption, smoking, and consumption of sweet tasting soft drinks; cluster 5 (14.18%): less salt and oil intake and lack of physical activity; cluster 6 (7.85%): no use of dairy products; cluster 7 (9.08%): the most unhealthy lifestyles; excessive work-related physical activity and smoking and unhealthy diet. Male gender was associated with higher odds of being in clusters 4 and 7. Individuals who were in unhealthy lifestyle clusters were mostly less educated and more self-employed or laborers.

**Conclusions:**

A very small percentage of individuals was in the healthy lifestyle cluster yet they had poor nutrition. Health policy-makers should pay more attention to low recreational physical activity among elder people and in middle-aged and housekeepers, and also to high work-related physical activities that have a strong tendency to be in a cluster with smoking among workers and less educated men.

Among the main causes of non-communicable diseases (NCD), lifestyle risk factors such as unhealthy diet, tobacco use, and lack of physical activities are the most important causes of cardiovascular diseases, type 2 diabetes, hypertension, and cancer. According to the World Health Organization (WHO) report, the prevalence of these risk factors is increasing worldwide (1-4). Additionally, a large proportion of mortality and disease burden is attributed to lifestyle risk factors (2, 5). In recent years, researchers have tended to cluster lifestyle factors. They believe that these risk factors are not randomly distributed in the population and occur in combination with other lifestyle risk factors. Clustering of lifestyle risk factors is associated with higher risk of different diseases that can be expected from each of the risk factors alone. Since the existing lifestyle clusters in a community can be associated with different patterns of demographic and social risk factors (6, 7), identification of different lifestyle patterns and the related factors in the country can be useful to find the high risk subgroups for appropriate interventions. Therefore, mere knowledge of the prevalence of each lifestyle risk factor in the national level is not enough for planning purposes.

It is essential to identify the lifestyle patterns in each country. Although there are many published papers on the prevalence of lifestyle risk factors in Iran, we could not find any studies on clustering of these factors (1, 6, 8-9). To the best of our knowledge, no study has evaluated lifestyle patterns in a national level in Iran. The objective of the present study was to determine the lifestyle patterns and the related risk factors in the Iranian population.

## Methods

The present study was performed using the most recent data obtained from the national survey of risk factors of non-communicable disease (SuRFNCD). The details of the methodology of this study have been published elsewhere (10). Briefly, the SuRFNCD was a population-based cross-sectional study on a representative sample of Iranian population. A multistage cluster sampling design was used in each province and 12000 subjects aged 6 to 70 years old were surveyed from May 22 and June 20, 2011 by the WHO Stepwise approach. Finally, after cleaning the data, 8244 subjects aged 25 to 70 years were analyzed in the report. 

**Measurements: **The participants have been interviewed during household visits by trained interviewers using the WHO stepwise standard questionnaire for NCD risk factors surveillance. Informed consent was obtained before conducting the interviews. The data including sex, age, education, occupation as well as the data related to lifestyle behaviors such as diet, physical activities, and tobacco and alcohol consumption were collected.

*The Global Physical Activity Questionnaire* *was used for assessing physical activity in 3 main domains of work-related activity, recreational activity, and walking (*11*). *The questions about diet were designed based on the WHO guidelines for chronic diseases risk factor surveillance. Two questions were used for estimating the mean serving of fruits, vegetables, and dairy products per day. The first question was related to the frequency of consuming fruits, vegetables or dairy products in a typical week (as how many days per week) and the second question was about the mean number of servings consumed in those days. Using these two questions, we could calculate the mean number of servings consumed per day (12). Furthermore, one question addressed the frequency of consuming fast foods or sweet-tasting soft drinks per week (like coke, or other sugary soft drinks, not fruit juices). We used the mean frequency of consuming fast foods and sweet-tasting soft drinks per week in our analysis. In the present study, there was no question about the amount of salt and oil consumption; however, there were two questions as “Do you add salt to your food at the table?” and “What kind of oil do you usually use?” These two questions were used as surrogates of the amount of oil and salt consumption. 

As for consuming oil, the participants were categorized into 2 groups: 1) people who mostly consumed saturated oil (hydrogenated vegetable fats, butter or other animal fats, margarine) and 2) those who mainly consumed unsaturated oil (vegetable oil or frying oil). The following questions were used to evaluate the status of smoking, and alcohol and tobacco use: “Do you smoke any kind of cigarette at this time? (Any type of factory-made cigarettes, hand-made cigarettes, or cigars)”, “Do you use the hookah at this time?” and “Did you drink alcoholic beverages in the past year?”

**Statistical analysis: **The mean and standard error were reported for quantitative variables, and frequency and percentage were used for qualitative variables. About 3.32% of the cells had missing data. Missing data were imputed performing single imputation and regression model in the MICE package in the R software (13-14). The self-organizing map (SOM) was applied for clustering (15-18). SOM is a nonparametric and single-layer artificial neural network clustering technique to group similar individuals (the details of this method are reported in appendix A). The silhouette index was used to decide the optimum number of clusters (19). 

Continuous variables fit better in these methods, so we used principal component analysis (PCA) for converting binary variables into continuous variables (22). In this study, 5 binary variables (salt, oil, smoking, alcohol, and hookah) were converted into 5 continuous variables by PCA, and the 5 PCA components were used for clustering and calculating the ICC instead of 5 binary variables. However, we referred to the original binary variables for interpretation of the variables in each cluster.

After cluster analysis, all further analyses were done with complex survey methods to control cluster sampling, stratification, and sampling weight. The data were weighted based on the 2011 national Iranian population aged≥ 25 and ≤ 70 years. First, the weighted prevalence and means with corrected 95% confidence interval were used to report the lifestyle behaviors and participants’ characteristics in each cluster. Second, multinomial logistic regression analysis with a complex survey design was used to determine the association between demographic characteristics and lifestyle clusters. All cluster analyses were performed using the R statistical package and all further analyses were performed using the STATA (12 version) for complex survey analysis.

## Results

Overall, 8244 individuals with a mean age of 42.21±13.60 years (standard error =0.03) were enrolled in the present study. Of these, 3305 (40.1%) were men and 2446 (29.67 %) were from rural areas. The weighted mean consumption of fruits, vegetables, and dairy products was 1.65, 0.92, and 1.17 units per day, respectively. The weighted mean consumption of fast foods and sweet-tasting soft drinks was 0.38 and 1.64 times per week, respectively. Approximately 54.46% of the participants added salt to their meals and 41.66 % usually used saturated fats. Table 1 presents other findings.

**C** **luster** **analysis: **The results of SOM clustering showed that 7 lifestyle behavior clusters were identified (silhouette index was used to decide the optimum number of clusters. The lifestyle-related characteristics of each group are shown in table 2. The characteristics of each group are as follows.

**Class 1: Healthy lifestyle behavior group (15.84%): **This group included an equal number of middle-aged men and women with a mean age of 37.83 years who had higher mean consumption of fruits, vegetables, and dairy products than other groups. They also consumed less sweet-tasting soft drinks, salt, saturated oil, and fast food than other groups. Besides, in comparison with other groups, they have the highest mean recreational physical activity (1.6 hours per week). On the contrary, they did not use tobacco at all.

**Class 2** **: Unhealthy lifestyle behavior group (with high consumption of fast foods, salt, and sweet-tasting soft drinks, 12.45%): **This group was a mixture of younger men and women (mean age = 38.46 years). A dominant feature of this group was that they did not use tobacco, but their recreational physical activity was almost zero. On the other hand, they consumed much more salt, sugary soft drinks, and fast foods than other groups. This group has higher education and the most employee people after class 1. 

**C** **lass** **3** **: Unhealthy lifestyle behavior group (with low physical activity, 33.73%): **Most of the participants in this group were housewives with no recreational physical activity. Besides, the consumption of fruits and vegetables

was very low, but they did not eat fast foods.

**C** **lass** **4** **: Unhealthy lifestyle behavior group (with smoking and consumption of alcohol and sweet-tasting soft drinks, 6.86%): **Although they had suitable work-related or recreational physical activity, they had poor eating habits and the use of tobacco and alcohol was high among them. In addition, they consumed a lot of salt and sweet-tasting soft drinks.

**C** **lass** **5** **: Unhealthy lifestyle behavior group (without any physical activity and low salt consumption, 14.18%): **This group was composed of elderly people with an older mean age than other groups. They consumed less salt and unsaturated fat. However, they almost had no work-related or recreational physical activity. 

**C** **lass** **6** **: Unhealthy lifestyle behaviors group (with unhealthy diet, low physical activity, no use of dairy products, 7.85%): **They did not use salt and dairy products and had no recreational physical activity, although they accepted physical activity at work and walking. Fast food consumption in this group was almost zero and they used a lot of saturated oil.

**C** **lass** **7** **: Unhealthy lifestyle behaviors group (with considerable smoking and work-related physical activity, 9.08%): **These middle-aged men only used cigarette among different tobacco products and had vigorous work-related physical activity and poor nutrition, seem to be male laborers who smoke a lot. People in this group have the unhealthiest lifestyles. According to the results in table 2, clusters 5, 7 had the highest mean age in order. There was an equal number of men and women in cluster 1. In clusters 4 and 7, approximately 81.62% and 95.61% of the participants were men, respectively. The rest of the demographic characteristics of each cluster is shown in more detail in table 2. 

Table 3 shows the results of the relationship between demographic characteristics and individuals in each cluster using a multinomial logistic model. Age was associated with individuals in clusters 5 and 7, and male gender was associated with higher odds of individuals in clusters 4 and 7. 

Living in urban or rural areas had no significant association with clusters. Evaluation of the relationship between education and occupation showed that individuals who were in unhealthy lifestyle clusters were mostly less educated and more self-employed or workers. The results are presented in table 3 in more detail.

**Table 1:** Description of the classes of lifestyle behaviors in the general population using self-organizing map among the Iranian population in 2011.

**N (percent) **	**Class 1** 1	**Class 2 ** 2	**Class 3 ** 3	**Class 4 ** 4	**Class 5 ** 5	**Class 6 ** 6	**Class 7 ** 7
	1306 (15.84)	1026 (12.45)	2781 (33.73)	566 (6.86)	1169 (14.18)	647 (7.85)	749 (9.09)
Nutrition	1.87(0.04)	1.05(0.04)	1.37(0.02)	1.10 (0.05)	0.91 (0.03)	0.38 (0.03)	0.47 (0.02)
Mean serving of vegetables in a day	1.86 (0.05)	0.58 (0.03)	1.07(0.05)	0.39 (0.03)	0.83 (0.07)	0.35 (0.04)	0.31 (0.03)
Mean serving of dairy products in a day	2.39 (0.1)	1.56 (0.08)	2.03(0.07)	0.93 (0.05)	1.69 (0.08)	0	1.25 (0.06)
Mean number of days per week for fast foot	0.27 (0.03)	1.57 (0.05)	0.01(0.00)	0.82 (0.07)	0.02 (0.00)	0	0.62 (0.05)
Mean number of days per week for sweet-tasting soft drinks	0.94 (0.04)	3.15 (0.06)	0.99(0.03)	3.19 (0.10)	0.78 (0.04)	1.29 (0.04)	1.80 (0.07)
Adding salt to food (percent)*	519(40.74)	961 (92.66)	1335 (48)	345(62.95)	439 (37.55)	250(37.64)	489(68.29)
unsaturated oil (percent) *	372(28.48)	435 (42.40)	1183(42.54)	277(48.94)	397 (33.96)	470(72.64)	305(40.72)
**Physical activities **							
Mean number of hours a week for work related physical activity	1.63 (0.12)	1.68 (0.13)	0.62 (0.08)	1.92 (0.09)	0.24 (0.04)	1.23 (0.01)	3.42 (0.21)
Mean number of hours a week for recreational physical activities	1.65 (0.08)	0.01 (0)	0	0.52 (0.07)	0.02 (0.01)	0.02 (0.01)	0.34 (0.04)
Mean number of hours a week for walking	0.74 (0.04)	0.54 (0.04)	0.35 (0.03)	0.57 (0.04)	0.13 (0.02)	0.43 (0.03)	0.79 (0.04)
**Tobacco**							
Cigarettes (percent) *	1 (0.00)	0	0	184(34.51)	0	0	749 (100)
Hookah (percent) *	0	0	0	259 42.76)	0	0	0
Alcohol (percent) *	0	0	0	336(60.29)	0	0	0

1 Healthy lifestyle behaviors group

2 Unhealthy (with high consumption of fast foods, salt, and sweet-tasting soft drinks)

3 Unhealthy (with low physical Activity)

4 Unhealthy (with smoking and consumption of alcohol and sweet-tasting soft drinks)

5 Unhealthy (without any physical activity and low salt consumption)

6 Unhealthy (with unhealthy diet, low physical activity, no use of dairy products)

7 Unhealthy (with considerable smoking and work-related physical activity)

* values are reported as with frequency and weighted percentage and for others weighted mean and standard error are reported (Data were weighted based on the 2011 national Iranian population aged≥ 25 and ≤ 70 years).

**Table 2:** Sociodemographic characteristics of the seven classes of lifestyle behaviors among the Iranian population in 2011.

**Variables **	**Class** **1**^1^	**Class** **2 **2	**Class** **3 **3	**Class** **4 **4	**Class** **5 **5	**Class** **6 **6	**Class** **7 **7
Age*	37.83 (0.34)	38.46 (0.47)	39.16 (0.15)	39.55 (0.54)	65.67 (0.07)	39.13 (0.43)	43.91 (0.47)
Sex (men)	617 (52.66)	367 (41.00)	648 (29.02)	423 (81.62)	340 (32.86)	215 (38.26)	695 (95.61)
Region (urban)	1005 (76.67)	749 (74.82)	1939 (69.53)	382 (67.12)	803 (69.53)	387 (58.68)	533 (69.75)
Education							
<6 years	600 (44.51)	641 (60.50)	1848 (63.07)	407 (61.74)	1070 (88.91)	509 (73.04)	562 (71.39)
6- 12 years	362 (27.27)	229 (23.09)	585 (22.36)	101 (30.02)	67 (6.05)	95 (18.52)	133 (21.05)
>12 years	344 (28.22)	156 (16.32)	348 (14.57)	58 (9.24)	32 (5.04)	43 (8.44)	54 (7.56)
Job							
Employee	222 (18.80)	98 (12.04)	200 (9.17)	37 (5.41)	3 (0.44)	31 (6.76)	56 (8.42)
Worker	75 (6.30)	50 (6.79)	103 (8.14)	151 (26.56)	21 (2.56)	55 (11.96)	87 (11.36)
Self-employed	241 (19.62)	195 (22.57)	379 (16.61)	234 (40.32)	125 (17.33)	107 (19.92)	352 (45.20)
Housewife	508 (36.46)	564 (46.38)	1764 (51.42)	38 (7.63)	771 (47.13)	379 (48.41)	38 (6.07)
Others	260 (18.82)	119 (12.22)	335 (14.67)	106 (20.08)	249 (32.53)	75 (12.94)	216 (24.22)

1 Healthy lifestyle behaviors group

2 Unhealthy (with high consumption of fast foods, salt, and sweet-tasting soft drinks)

3 Unhealthy (with low physical Activity)

4 Unhealthy (with smoking and consumption of alcohol and sweet-tasting soft drinks)

5 Unhealthy (without any physical activity and low salt consumption)

6 Unhealthy (with unhealthy diet, low physical activity, no use of dairy products)

7 Unhealthy (with considerable smoking and work-related physical activity)

* Age is reported as with weighted mean (SE) and the rest of the variables are reported with unweighted frequency and weighted percentage (Data were weighted based on the 2011 national Iranian population aged≥ 25 and ≤ 70 years).

**Table 3:** Association between sociodemographic characteristics and lifestyle behavior patterns using a multinomial logistic model, weighted odds ratios with 95% confidence intervals (among the Iranian population in 2011).

**Variables**	**Class 2**	**Class 3**	**Class 4**	**Class 5**	**Class 6**	**Class 7**
Age	0.98 (0.97-1.02) (p=0.098)	0.99 (0.97-1.01) (p=0.336)	0.97 (0.96-1.00) (p=0.107)	1.47 (1.53-1.62) (p<0.0001)	1.00 (0.98-1.01) (p=0.894)	1.02 (1.01-1.03) (p<0.0001)
Sex (men)	0.83 (p=0.378)	0.33 (0.25-0.44) (p=0.040)	2.28 (1.42-3.12) (p<0.0001)	0.58 (0.30-0.88) (p=0.029)	0.89 (0.37-1.64) (p=0.107)	3.28 (2.60-7.72) (p<0.0001)
Region (urban)	0.88 (0.57-1.35) (p=0.575)	1.02 (0.85-1.46) (p=0.401)	0.93 (0.60-1.46) (p=0.778)	0.91 (0.81-1.89) (p=0.300)	1.16 (0.96-2.68) (p=0.079)	0.71 (0.57-1.32) (p=0.656)
Education category						
>12 years	1	1	1	1	1	1
6- 12 years	1.29 (0.96-1.72) (p=0.082)	1.65 (1.33-2.05) (p<0.001)	1.62 (0.88-2.77) (p=0.113)	1.46 (0.717-2.98) (p=0.288)	1.80 (1.08-3.33) (p=0.025)	2.45 (1.54-3.91) (p<0.0001)
<6 years	1.32 (1.43-2.75) (p<0.043)	2.55 (1.01-3.23) (p<0.001)	4.45 (2.43-6.13) (p<0.0001)	3.73 (1.78-7.83) (p=0.001)	3.84 (1.85-7.22) (p<0.0001)	5.33 (4.93-12.08) (p<0.0001)
Job category						
Employee	1	1	1	1	1	1
Worker	1.12 (0.67-1.86) (p=0.648)	1.07 (0.73-1.58) (p=0.713)	1.42 (1.03-2.99) (p=0.042)	1.14 (0.85-4.92) (p=0.316)	2.08 (1.07-3.05) (p=0.030)	1.79 (1.04-3.07) (p=0.034)
Self-employed	1.36 (0.90-2.06) (p=0.138)	1.30 (1.04-1.72) (p=0.0113)	2.44 (1.48-3.03) (p=0.001)	2.20 (1.15-5.51) (p=0.004)	1.30 (0.67-2.52) (p=0.427)	1.85 (1.23-2.78) (p=0.004)
Housekeeper	1.46 (1.12-2.56) (p=0.017)	2.41 (0.85-1.34) (p=0.002)	0.94 (0.23-3.66) (p=0.243)	1.81 (1.23-11.04) (p=0.061)	1.16 (0.59-2.28) (p=0.647)	0.26 (0.19-1.62) (p=0.123)
Others	0.97 (0.59-1.58) (p=0.335)	1.09 (0.79-1.50) (p=0.585)	1.29 (0.78-2.73) (p=0.131)	2.87 (1.86-10.96) (p=0.002)	1.16 (0.67-2.00) (p=0.583)	1.54 (0.86-2.75) (p=0.135)

## Discussion

To the best of our knowledge, the present study was the first to show a snapshot of the lifestyle patterns in our country. According to the results, the study population could be categorized in 7 sub-groups in terms of lifestyle variable. Investigation of the specific characteristics of these 7 groups showed several important and interesting findings. The most important finding of this study was that only 16% the population were in the healthier lifestyle cluster and about 84% of the population were in other clusters with some unhealthy lifestyle behaviors; yet, those in the healthy lifestyle cluster used fruits, vegetables and dairy products less than standard recommendations. In fact, it seems that no group in the country has a healthy lifestyle according to standards. Similar studies in different countries have shown different results; for instance, a study conducted in the Netherlands showed that 80% of the population were in the healthy lifestyle cluster (23) or another study conducted in France showed there were 6 lifestyle clusters in the country and 41% of the population were in healthy lifestyle clusters (24). Another important finding of this study is that although the mean physical activity in the country, according to standard definitions, is not very low (at least 20 minutes three times a week), it is mostly related to work or limited to walking. Except group 1, recreational physical activity in other groups was almost zero and mostly comprised middle-aged and women housekeepers and elderly population, which warrants further attention. However, more interventions are required to increase recreational physical activities. Furthermore, the largest cluster comprised individuals who did not have recreational physical activity and were mostly women and elder population.

Nevertheless, individuals in clusters 4 and 7 used a lot of tobacco and had considerable work-related physical activity; moreover, they had some recreational activity (physical exercise), as well. A high percentage of people in these two groups were self-employed and were undergraduates. It seems that work-related physical activity and smoking tend to be in one cluster. So, care should be taken to not ignore people who have a good level of total physical activity because many of them are smokers and have poor nutrition, requiring intervention specially among men with less education and more self-employed or workers labors. 

This finding is inconsistent with the results of many other studies. A large number of studies on lifestyle clustering have shown that smoking and no or little physical activity tend to be in a cluster (9, 17-18). It should be noted that in similar studies, work-related and recreational physical activities were not separated, which could be one of the reasons for this difference. An inverse relationship may be observed in countries with a higher ratio of recreational to work-related physical activity (17, 25).

It seems this study has defined work-related and recreational physical activity in lifestyle clustering independently for the first time. Several studies have shown that work-related or recreational physical activity may have different effects on human health. For example, some studies have revealed that increased recreational physical activity reduces the risk of cardiovascular diseases while increased work-related physical activity is associated with increased risk of cardiovascular diseases (26-28). The findings of similar studies in this area suggest that work-related and recreational activities should be examined separately (29. Yet, this point is usually neglected in studies related to lifestyle clustering but in the present study, we decided to separate these two kinds of physical activities for their different health effects.

The results of the present study showed that unhealthy lifestyle clusters with risk factors like tobacco and alcohol use were more frequent in men and in less educated people. This finding is similar to the results of various studies in the world. Most studies have shown that male gender and low education are more related to unhealthy lifestyle clustering (9, 17, 23). People in cluster 5 had the highest mean age than the other groups. In fact, the members of this group were older people who used less salt and oil than other groups, which could be due to interventions. Though, the consumption of dairy products, fruits, and vegetables was very low in this group, and most importantly, the mean physical activity was very low. The fact that most members of this group were retirees justifies the lack of work-related physical activity, but very low recreational physical activity and even walking should be noted in this group. According to many studies, important reasons for the lack of physical activity in older people include the high cost of sports, lack of energy, and transportation-related barriers in this group of people (30-32). Therefore, one of the most important interventions in this group is emphasized on physical activity and provision of suitable conditions in urban and rural areas along with interventions to promote healthy eating habits. 

One of the most important strong points of the present study is that recreational and work-related physical activities were defined separately because they may have different effects on the human health. One of the main limitations of this study is the high number of missing. Although we tried to replace them using imputation methods, it could still affect the results. Another limitation is the lack of accurate information on some variables such as marital or economic status, which we could not measure. The data related to the amount of salt and oil consumption were not available. Thus, two variables, i.e. adding salt to food during serving and the type of oil, were used as substitutes. 

In conclusions the results of this study showed that in Iran, like many other countries, lifestyle risk factors tend to be in cluster and create different subgroups. Nonetheless, a remarkable point is that a very small percentage of people were in clusters with healthy lifestyles and this small percentage had poor nutritional habits. It should be noted that high work-related physical activities have a strong tendency to be in a cluster with other unhealthy lifestyle factors especially smoking among workers and less educated men. As a result, it is necessary to pay special attention to interventions related to decreasing smoking in this group. 

Furthermore, the largest cluster of unhealthy lifestyle belonged to people who did not have any recreational physical activity and mostly comprised middle-aged and women housekeepers and elder population. Consequently, we need to apply interventions to increase recreational physical activities in these groups of people. 
